# Antinociceptive effects, acute toxicity and chemical composition of *Vitex agnus-castus* essential oil

**Published:** 2015

**Authors:** Emad Khalilzadeh, Gholamreza Vafaei Saiah, Hamideh Hasannejad, Adel Ghaderi, Shahla Ghaderi, Gholamreza Hamidian, Razzagh Mahmoudi, Davoud Eshgi, Mahsa Zangisheh

**Affiliations:** 1 *Division of Physiology, Department of Basic Science, Faculty of Veterinary Medicine, University of Tabriz, Tabriz, I.R.Iran. *; 2 *Division of Histology, Department of Basic Science, Faculty of Veterinary Medicine, University of Tabriz, Tabriz, I.R.Iran.*; 3 *Department of Food Hygiene and Aquatics, Faculty of Veterinary Medicine, University of Tabriz, Tabriz, I.R.Iran.*

**Keywords:** *Vitex agnus-castus Antinociception*, *Acute toxicity*, *Chemical composition*, *Rat*

## Abstract

**Objective:**

*Vitex agnus-castus* (VAC) and its essential oil have been traditionally used to treat many conditions and symptoms such as premenstrual problems, mastalgia, inflammation, sexual dysfunction, and pain. In this study, the effects of essential oil extracted from *Vitex agnus-castus* (EOVAC) leaves were investigated in three behavioral models of nociception in adult male Wistar rats.

**Materials and methods:**

Chemical composition of EOVAC was analyzed using gas chromatography – mass spectrometry (GC-MS) and also its possible toxicity was determined in mice. Analgesic effect of EOVAC was determined using tail immersion test, formalin test, and acetic acid-induced visceral pain in rats.

**Results:**

EOVAC (s.c.) and morphine (i.p.) significantly (*p*<0.05) reduced pain responses in both formalin and tail immersion tests. In the study of evolved mechanisms, pretreatment with naloxone or atropine significantly (*p *<0.05) reversed the essential oil-induced analgesia in both formalin and tail immersion tests. Moreover, EOVAC and Piroxicam produced significant (*p<0.05*) inhibition in the acetic acid-induced writhing response. EOVAC did not show any mortality even at high dose (5 g/kg, p.o.) of administration in toxicity test. Moreover, according to GC-MS results, major components of the EOVAC were α-pinene (14.83%), limonene (10.29%), β-caryophyllene (6.9%), sabinene (5.27%), and β-farnesene (5.9%).

**Conclusions:**

These results suggest that endogenous opioidergic system as well as muscarinergic receptors of cholinergic system may be involve in the antinociceptive activity of *Vitex agnus-castus* essential oil in these models of pain in rats.

## Introduction

*Vitex agnus-castus* (VAC) is a small deciduous shrub commonly known as monk pepper or chaste tree belonging to the Lamiaceae family of plants that is widely distributed in the Middle East and Mediterranean region (^Stojkovic' et al., 2011 ▶^). VAC is traditionally used as a treatment for menstrual problems, inflammation, sexual dysfunction, and pain (Upton, 2001). In the Iranian folk medicine, VAC is used as anticonvulsant, antiepileptic, carminative, energizer, sedative, anticonvulsant, constipation, and reduction of libido (Nasri and Ebrahimi, 2006 ▶; Saberi et al., 2008 ▶; Ramazani et al., 2010 ▶; Safa et al., 2012 ▶). 

Different kind of extracts from VAC have been reported to produce antinociceptive and anti-inflammatory effects (Ramazani et al., 2010 ▶), enhance female fertility (Dugoua et al., 2008 ▶), and reduce moderate to severe symptom of premenstrual syndrome (PMS) such as mastalgia, headache, fatigue, anxiety, and depression (Atmaca et al., 2003 ▶; Prilepskaya et al., 2006 ▶). Moreover, essential oil of VAC has shown anti-microbial and anti-fungal activities (Choudhary et al., 2009 ▶**; **Ghannadi et al., 2012 ▶). 

Essential oil is a volatile aromatic compound from plants that have been used medicinally throughout history (Christaki et al., 2012 ▶). EOVAC contains some important monoterpenes and sesquiterpenes such as α-pinene, α-bisabolol, 1,8-cineol, β-caryophyllene, and limonene (^Stojkovic' et al., 2011 ▶^; Ghannadi et al., 2012 ▶ ). Previous studies have indicated that some of these terpenes have anti-inflammatory and antinociceptive effects in different models of pain and inflammation (Guimarães et al., 2013 ▶). There are some other monoterpenes in the EOVAC such as α-phellandrene and Linalool. It has been shown that both α-phellandrene and linalool could produce analgesia via cholinergic and opioidergic systems in different models of pain in the rodents (Peana et al., 2003 ▶; Lima et al., 2012 ▶). 

Beneficial effect of VAC extracts in the treatment of PMS symptoms has caused an increasing interest for determination of its possible mechanisms of action in PMS symptoms. Moreover, recently, Webster et al., (2011) ▶ reported that therapeutic effects of different fraction of VAC extract are mediated through the activation of µ and δ but not κ opioid receptors. 

Despite the demonstration of the efficacy of VAC extracts in the treatment of PMS symptoms and reduction of pain perception, nothing has been published about the effects of EOVAC in pain modulation. Therefore, the present study was aimed to investigate the antinociceptive activity of EOVAC on the chemical, thermal, and inflammatory models of pain. Moreover, we used naloxone (nonselective opioid receptors antagonist) and atropine (nonselective muscarinic receptors antagonist) to determine its possible opioidergic or cholinergic mechanisms of action in these models of pain. 

The content and composition of extracted essential oils from one plant species vary in different seasons, soil component, and weather conditions (D'Antuono et al., 2000 ▶). Because of these reasons, our extracted essential oil was analyzed using GC-MS to determine its active ingredients. 

## Materials and Methods

**Animals**

Adult male Wistar rats, weighing 250-280 g and adult male Swiss albino mice, weighing 20-25 g of were used in this study. They were randomly housed in polyethylene cages with *ad libitum* access to food and water in a room with controlled temperature (22±1 °C) and under a 12 h light–dark cycle (lights on from 07:00 h). Seven, Six, and five rats were used in each group of tail immersion, formalin, and writhing tests, respectively. All experiments were performed between 11:00 h and 15:00 h. All research and animal care procedures were approved by the Veterinary Ethics Committee of the Faculty of Veterinary Medicine, (University of Tabriz), Iran and were performed in accordance with the current guidelines for the care of laboratory animals and the ethical guidelines for investigations of experimental pain in conscious animals (Zimmermann, 1983 ▶).

**Drugs and chemicals**

Morphine sulfate was purchased from Tolid Darou Co. (Tehran, Iran). Atropine and naloxone hydrochloride, piroxicam and Tween 80 were purchased from Sigma Chemical Co. (St. Louis, MO, USA) and formalin solution 37% was purchased from Merck Chemicals (Darmstadt, Germany). Acetic acid solution 99.5% was purchased from Dr. Mojallali Chemicals Co. (Teheran, Iran). All drugs and chemicals were dissolved in physiological saline. An emulsion of essential oil was prepared using Tween 80 and saline (2%, v/v) as solvent. 

**Plant material and essential oil extraction**

The leaves of *Vitex agnus-castus* was collected during August - September in 2012 from vicinity of Maragheh county in the East Azerbaijan, Iran and were subsequently authenticated by Dr Fatemeh khoshbakht Koolagh, a botanist at the Herbarium of Faculty of agriculture, University of Tabriz, Tabriz, Iran. A voucher specimen (16697) was deposited at the Herbarium of Faculty of agriculture. The leaves were dried in room temperature avoiding from direct sunlight and then ground into a fine powder. 

The essential oil of *Vitex agnus-castus* leaves were extracted from powdered plant by hydrodistillation in a Clevenger type apparatus for 4 h and produced 0.7% (v/w) yield. Obtained essential oil was dried over anhydrous sodium sulfate until the last traces of water were removed, and then stored in dark glass bottles at 4 °C (Mahmoudi et al., 2014 ▶).

**Nociceptive tests**

*Formalin Pain*

For reduction of possible effect of stress during the test, on three successive days prior to the formalin test, animals were placed for 30 min inside a Plexiglas observation chamber (30×30×25 cm^3^) equipped with a mirror angled at 45° below the chamber (Abbott and Bonder, 1997 ▶; Khalilzadeh et al., 2010 ▶). In the test day after a 30-min adaptation period, formaldehyde solution (2.5% in saline, 50 µl/paw) was injected subcutaneously into the ventral surface of the right hind paw using a 30-gauge injection needle (Khalilzadeh et al., 2010 ▶).

Following formalin injection, the rat was immediately put back into the opaque observation chamber. The time spent in licking and biting of the injected paw determined as a nociceptive behavior and was recorded in uninterrupted 5-min blocks over a period of 45 min. The first 5 min measured as the first phase (phasic pain, neurogenic phase) while the period between 15 - 45 min was considered as the second phase (inflammatory pain) (Khalilzadeh et al., 2010 ▶). 

*Tail Immersion Test*

The tail of rats was immersed (5cm) in a water bath at noxious temperature of 55±0.5 °C until the tail was withdrawn or the whole body was recoiled. The cut-off time was fixed at 15 s to prevent any tissue damage to the tail (Le bars et al., 2001 ▶). Reaction latencies at 0, 15, 30, 45, 60, and 90 min after chemicals administration were used as a parameter reflecting of the pain experienced. 

*Treatment groups in formalin and tail immersion tests*

Group 1: This group received s.c. injection of vehicle (Tween 80, 2% v/v in saline, 200 µl) before intraplantar injection of formalin or tail immersion test. 

Groups 2, 3, 4, and 5: In these groups, s.c. injection of EOVAC at doses of 25, 37.5, 50, and 62.5 mg/kg, respectively, were performed before intraplantar injection of formalin or tail immersion test.

Groups 6, 7, and 8: In these groups, i.p. injection of saline (200 µl/rat), morphine (10 mg/kg), naloxone (1 mg/kg), andatropine (1 mg/kg), respectively were performed before intraplantar injection of formalin or tail immersion test.

Groups 9 and 10: These groups received i.p. injection of naloxone (1 mg/kg) and atropine at a dose of 1 mg/kg, respectively with s.c. injection of EOVAC (50 mg/kg) before intraplantar injection of formalin or tail immersion test.

The i.p. injections of naloxone and atropine were performed 40 min before intraplantar injection of formalin and 10 min before starting tail immersion test. The s.c. injections of EOVAC and i.p. injection of morphine were performed 30 min before intraplantar injection of formalin and 0 min (immediately after injection) before tail immersion test.

*Acetic acid induced writhing response in rat*

The test was carried out using the technique previously described by Tamaddonfard et al., (2008) ▶. For adaptation of animals to test environment, they were placed 30 min inside a Plexiglas opaque chamber (40×30×20 cm^3^) that equipped with a mirror angled at 45° below the chamber. Using a 27-gauge injection needle, Tween 80 (2%, s.c., 200µl) as control, EOVAC (25 and 50 mg/kg) and piroxicam (50 mg/kg) subcutaneously injected 30 min before intraperitoneal injection of acetic acid (2% v/v in saline, 4 ml/kg). Immediately after the injection of acetic acid, frequency of writhing response was recorded in 5-min periods during the test which lasted 40 min. Moreover, latency time to the beginning of the first abdominal contraction (first writhe) was recorded. A writhe was defined as a wave of the contraction of the abdominal musculature followed by extension of the hind limbs (Ness, 1999 ▶; Le Bars et al., 2001 ▶).

**Acute toxicity in mice**

For determination of chronic toxicity of essential oil, it was administrated at doses of 1000, 2000, 3000, and 5000 mg/kg/200 µl (p.o.) in four groups of mice (n=6 in each group). One group received an equal volume of Tween 80, 2% in normal saline as a solvent of essential oil. Animals' mortality was observed for 2 days. Food and water were provided ad libitum.

**Essential oil GC-MS analyze**

The essential oil was analyzed by gas chromatography–mass spectrometry (GC-MS). The chromatograph instrument (Agilent 6890, UK) was equipped with an HP-5MS capillary column (30 × 0.25 mm ID × 0.25 mm film thickness) and the data were taken under the following conditions: initial temperature 50°C, temperature ramp 5°C/min, 240°C/min to 300°C (holding for 3 min), and injector temperature at 290°C. The carrier gas was helium and the split ratio was 0.8 mL^-1^/min. For confirmation of analysis results, EOVAC was also analyzed by gas chromatography–mass spectrometry (Agilent 6890 gas chromatograph equipped with an Agilent 5973 mass-selective detector; Agilent UK) with similar capillary column and analytical conditions as above. The MS was run in electron-ionization mode with ionization energy of 70 eV (Mahmoudi et al., 2014 ▶). 

**Statistical analysis**

Data were analyzed using one-way analysis of variance (ANOVA) followed by Tukey’s HSD post hoc test for both formalin and writhing tests data and two-way analysis of variance (ANOVA) with repeated measures followed by Tukey's post hoc test was applied for determination of significance value in the tail immersion test using IBM^®^ SPSS^®^ software version 19 (IBM company, USA). Statistical differences between two control groups of formalin test were analyzed using student t-test. In figures, all values were expressed as mean ± SEM. A value of *p*<0.05 was considered as statistically significant**.**

## Results

**Tail immersion test**

**Figure 1:**
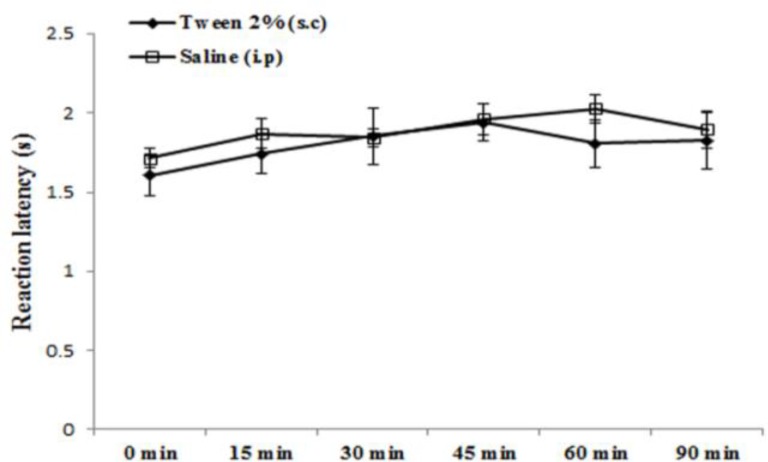
The effects of normal saline (200 µl, i.p.) and Tween 80 (2%, 200µl, s.c.) administration on withdrawal latency in rats. Values are expressed as the mean±SEM (n=7/group).

There were not significant differences between normal saline (200µl, i.p) and vehicle (Tween 2% v/v in saline, 200µl, s.c.) treated groups in the tail immersion test (Figure 1). 

**Figure 2:**
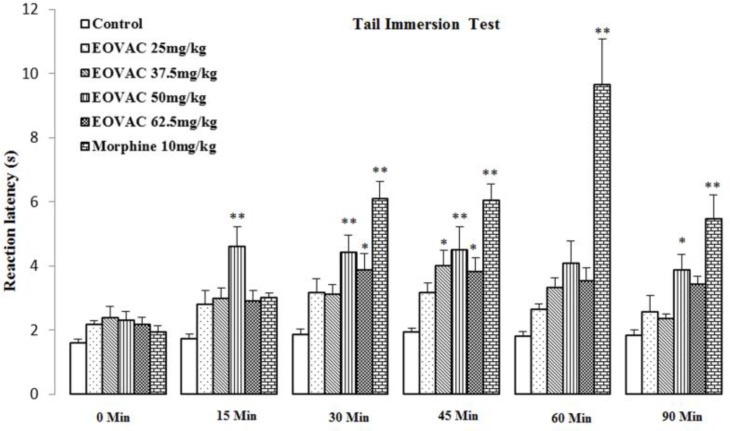
The effects of essential oil of *Vitex agnus-castus* and morphine on withdrawal latency in rats. * p<0.05 and ** p<0.001 as compared with the control group (n=7/group). (Two-way analysis of variance (ANOVA) with repeated measures followed by Tukey’s post hoc test), EOVAC: Essential oil of *Vitex agnus-castus*, Morph: Morphine, 0 min: Immediately after injection

Therefore, the obtained data from experimental groups were compared with vehicle treated group. Subcutaneous injection of 25 mg/kg of EOVAC failed to prolong withdrawal latency during the observation period. The effect of EOVAC (37.5 mg/kg, s.c.) on reaction latency was statistically significant (3.99± 0.49, *p*<0.05) at 45 min compared with the same time point of control value. The effect of EOVAC (50 mg/kg, s.c.) on reaction latency was statistically significant (4.60± 0.62, 4.42± 0.53, 4.51± 0.70, and 3.88± 0.47, *p*<0.05 and *p*<0.001) at 15, 30, 45, and 90 min, respectively compared with the same time point of control value. The effect of EOVAC (62.5 mg/kg, s.c.) on reaction latency was statistically significant (3.87± 0.51 and 3.81± 0.43, *p*<0.05 and *p*<0.001) at 30 and 45 min, respectively compared with the same time point of control value (Figure 2).

Morphine analgesic activity was started 30 min after i.p. injection which lasted until the end of observation period (*p*<0.001) (Figure 2).

**Figure 3:**
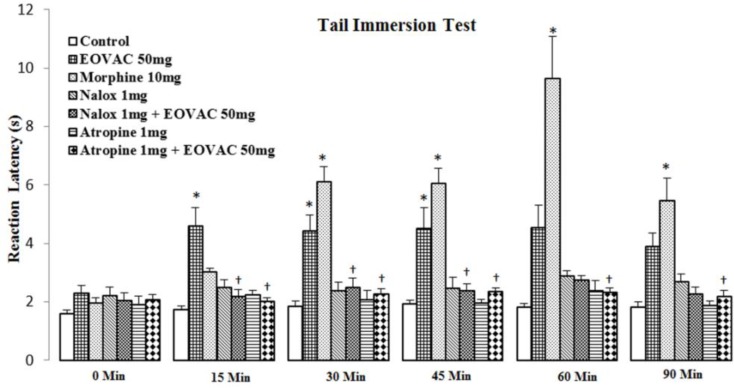
The effects of pretreatment with naloxone and atropine on *Vitex agnus-castus* essential oil induced antinociception in tail immersion test. * p<0.05 as compared with the control group.^ †^ p<0.05 as compared with the EOVAC 50 mg/kg treated group (n=7/group). (Two-way analysis of variance (ANOVA) with repeated measures followed by Tukey’s post hoc test). EOVAC: Essential oil of *Vitex agnus-castus*, Morph: Morphine, Nalox: Naloxone, Atrop: Atropine

Naloxone (1 mg/kg) and atropine (1 mg/kg) alone showed no significant effect but pre-treatment of animals with naloxone or atropine completely prevented (*p*<0.05) the EOVAC (50 mg/kg) analgesic activity during the observation period (Figure 3). 

**Formalin test**

**Figure 4:**
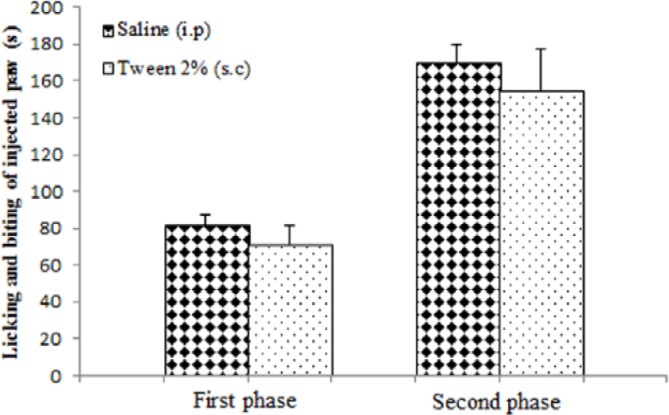
Formalin-induced nociceptive behavior (licking and biting) in normal saline (200 µl, i.p.) andTween 80 (2%, 200µl, s.c.) treated groups. Values are expressed as the mean±SEM (n=6/group

The intraplantar injection of the formalin 2.5% solution after vehicle (Tween 80, 2% v/v in saline, 200µl, s.c.) or saline (200µl, i.p.) produced nociceptive behavior in both the first (71±10.6 s and 81.5±5.94 s, respectively) and second phases (154.5 ± 22.7 s and 169.66±10.50 s, respectively) without any significant differences (Figure 4). 

**Figure 5:**
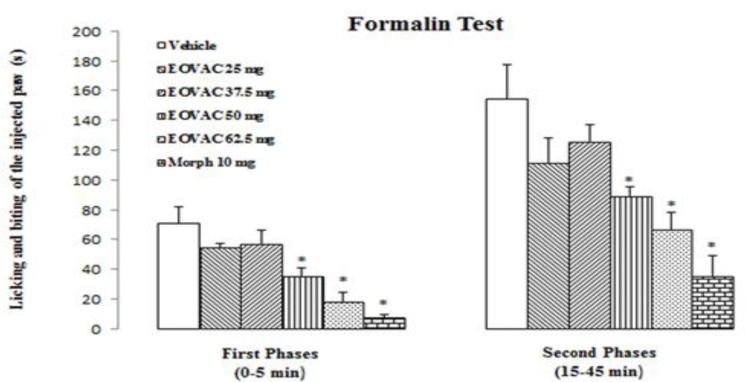
The effects of essential oil of *Vitex agnus-castus* and morphine on formalin pain response in rats. * p<0.05 as compared with control group (n=6/group). (One way ANOVA followed by Tukey’s HSD post hoc test). EOVAC: Essential oil of *Vitex agnus-castus*, Morph: Morphine

Therefore, the obtained data from experimental groups were compared with vehicle-treated group. EOVAC at doses of 25 and 37.5 mg/kg produced no significant effects in both first and second phase of formalin pain (Figure 5). 

The s.c. injection of EOVAC at doses of 50 and 62.5 mg/kg induced a significant (*p*<0.05) antinociceptive effect compared to the control group in the both first (35.83±4.81 s and 18.3±6.4 s, respectively) and second phases (89.3±5.8 s and 66.5±11.5 s, respectively) of the formalin test (Figure 5).

**Figure 6:**
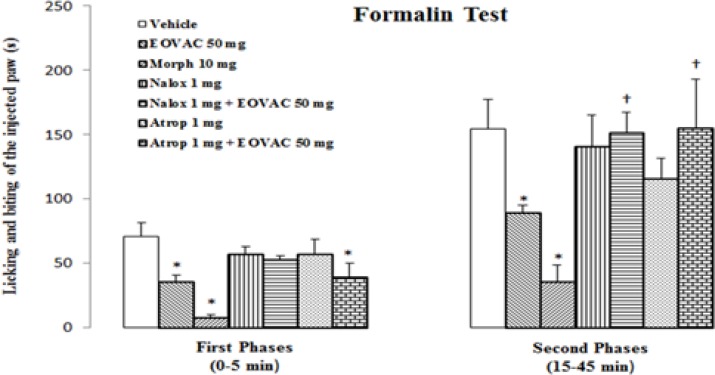
The effects of pretreatment with naloxone and atropine on essential oil of *Vitex agnus-castus* induced antinociception in formalin pain response. * p<0.05 as compared with control group.^ †^ p<0.05 as compared with EOVAC 50 mg/kg treated group (n=6/group). (One way ANOVA followed by Tukey’s HSD post hoc test). EOVAC: Essential oil of *Vitex agnus-castus*, Morph: Morphine, Nalox: Naloxone, Atrop: Atropine.

Morphine (10 mg/kg, i.p.) significantly inhibited nociception in the both first (7.5±2.1 s, *p*<0.05) and second phases (35.5±13.4 s, *p*<0.05) of formalin test. Opioid receptors antagonist naloxone (1 mg/kg) and non-selective muscarinic receptors antagonist atropine (1 mg/kg) alone had not any significant effect on both phases of formalin test (Figure 6).

Pre-treatment of animals with naloxone completely prevented the EOVAC (50 mg/kg) analgesic effect in the first (53±2.6 s, *p*<0.05) and second phase (151.6±15.4 s, *p*<0.05) of formalin test but atropine inhibited EOVAC analgesic effect only in the second phase (155.3±37.8 s, *p*<0.05) of formalin test (Figure 6).

**Acetic acid-induced writhing response in rat**

**Figure 7:**
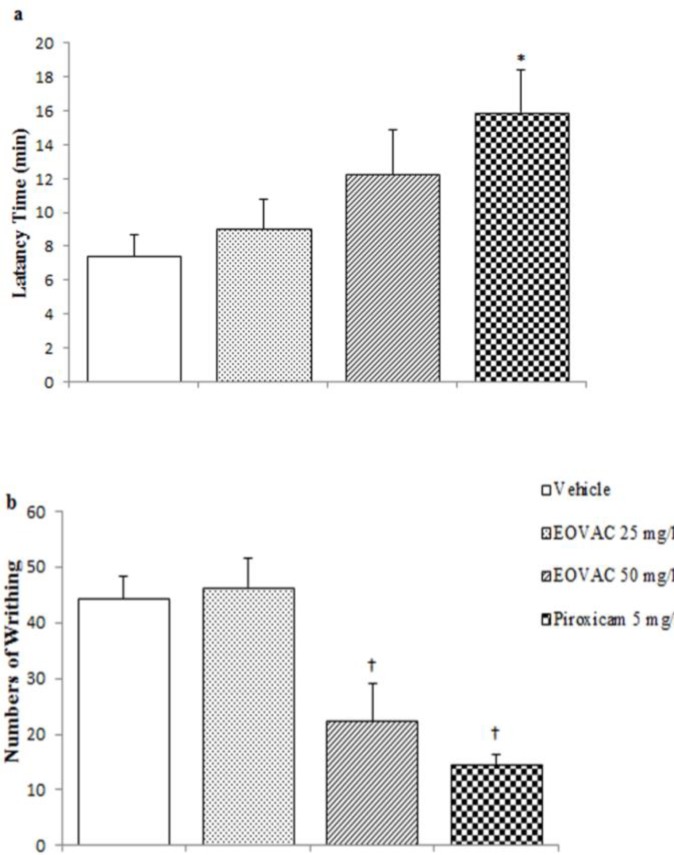
The effects of essential oil of *Vitex agnus-castus* and piroxicam on latency time to the beginning of the first writhe (a) and writhe numbers (b) in acetic acid-induced visceral pain in rat. * p<0.05, † p<0.01 as compared with control group (n=5/group). (One way ANOVA followed by Tukey’s HSD post hoc test). EOVAC: Essential oil of *Vitex agnus-castus*

The results presented in Figures 7A and 7B shows that EOVAC at doses of 50 mg/kg but not 25 mg/kg significantly (22.4±6.6 n,* p*<0.01) reduced number of abdominal writhes in comparison with control group (44.4±4 n, *p*<0.01) in the acetic acid induced visceral pain (Figure 7b). Administration of non-selective cyclooxygenase inhibitor (piroxicam, 5 mg/kg) significantly (14.4±1.9 n, p<0.01) reduced number of abdominal writhes and also significantly (15.88±2.47 min, p<0.05) increased latency time to the beginning of the first writhe in comparison with control group (7.3±1.3 min) (Figure 7A). EOVAC at dose of 50 mg/kg increased the latency time (12.28±2.5 min) to the beginning of the first writhe but this effect was not statistically significant (Figure 7a).

**Acute toxicity**

The essential oil of *Vitex agnus-castus *leaves showed no animal mortality even at dose of 5000 mg/kg in a period of 2 days. The LD_50_ value of this essential oil in mice is estimated to be more than 5 g/kg, (p.o.).

**GC-MS analysis**

The chemical composition of EOVAC was analyzed using GC-MS, which identified 22 compounds, representing 68.37% of total oil compounds. 

According to these results, major components of the EOVAC were α-pinene (14.83%), limonene (10.29%), β-caryophyllene (6.9%), sabinene (5.27%), and β-farnesene (5.9%). The major composition of EOVAC is represented in Table 1.

**Table1:** The major components (%) of *Vitex agnus-castus* leave essential oil

**N**	**Compound**	**RT** ^1^	**%**
1	α- Pinene	8.19	14.83
2	Sabinene	8.76	5.27
3	β- Myrcene	8.98	1.49
4	Phellandrene	9.18	0.52
5	α-Terpinene	9.38	0.67
6	Limonene	9.73	10.29
7	γ-Terpinene	10.00	0.85
8	α- Terpinolene	10.39	0.55
9	Linalool	10.51	0.86
10	α- Terpinenyl acetat	13.25	2.92
11	α- Bergamotene	13.62	2.19
12	Eugenol	13.36	1.71
13	β- Caryophyllene	14.07	6.9
14	β- Farnesene	14.20	5.9
15	α- Curcumene	14.47	1.4
16	Germacrene-D	14.56	2.26
17	β- Selinene	14.63	1.44
18	α- Aminomethylene glutaconic anhydride	14.75	1.32
19	β- Sesquiphellandrene	14.83	1.86
20	Caryophyllene oxide	15.43	1.58
21	β- Eudesmol	15.95	2.14
22	α- Bisabolol	16.1	1.42

## Discussion

In this study, three different nociceptive tests (formalin test, acetic acid-induced writhing response, and tail immersion test) were employed for evaluation of possible peripheral and central effects of the *Vitex agnus-castus* essential oil. Using these methods, it was revealed that subcutaneous injection of EOVAC produced antinociceptive effects in the rats.

Subcutaneous injection of formalin 2.5% into the ventral surface of right hind paw produced a biphasic pattern of nociceptive responses in rats. Each phase of formalin test has different mechanisms of nociception. The first phase is produced by direct simulative effect of formalin on myelenated and unmyelenated nociceptive afferent fibers, mainly C fibers which corresponds to acute nociceptive neurogenic pain (Shields et al., 2010 ▶). This phase of formalin pain is more sensitive to opioidergic agent’s effect. The second phase of formalin test is associated with release of several inflammatory mediators and excitatory amino acids such as glutamate and aspartate (Omote et al., 1998 ▶) causing an inflammatory type of nociception and is very sensitive to anti-inflammatory actions of non-steroid anti-inflammatory drugs as the cyclooxygenase inhibitors (Couto et al., 2011 ▶). Several studies suggested that the chemicals or drugs that act as analgesic via activation of central mechanisms of analgesia can inhibit both phases of formalin test whereas peripherally acting drugs can inhibit only the late phase (Yamamoto and Nozaki-Taguchi, 2002 ▶). Our results showed that EOVAC significantly reduced licking and biting behaviors in the both phases of formalin test and increased latency time in the tail immersion test at various time points post-treatment in rats. These findings suggested that the analgesic activity of EOVAC is mediated by both peripheral and central antinociceptive mechanisms in these models of nociception.

 In the present study, intraperitoneal injection of morphine (10 mg/kg) produced an inhibitory effect on both phases of formalin pain and also increased latency time in the tail immersion test. Moreover, pretreatment with naloxone (a non-selective opioid receptors antagonist), at a dose that did not produce any significant effect on the formalin pain or thermal pain responses, completely prevented analgesia induced by EOVAC on both phases of the formalin test as well as tail immersion test. These results indicated that at least part of the antinociceptive effect observed from EOVAC is due to activation of endogenous opioidergic system. 

Webster et al., (2011) ▶ reported that different fractions of *Vitex agnus-castus* extract act as an agonist of µ and δ but not κ opioid receptors. In addition, four days feeding of rats with VAC caused a significant increase in brain and blood levels of β-endorphin (endogenous opioid agonist) (Samochowiec et al., 1998 ▶). Opioidergic activity exhibited by VAC may be one of the important mechanisms of action of VAC in reduction of pain and treatment of PMS syndrome (Webster et al., 2011 ▶). 

In the present study, it was revealed that pretreatment with atropine (1 mg/kg) significantly prevented the analgesic effect of EOVAC (50 mg/kg) in the formalin test as well as tail immersion test. These results suggest an acetylcholine muscarinic receptors involvement in the analgesic effect induced by EOVAC. Cholinergic system has an important role in the pain modulation (Jones and Dunlop, 2007 ▶). EOVAC contains some of terpenes such as (-)-Linalool and α-phellandrene that produce analgesia via activation of cholinergic system (Peana et al., 2003 ▶; Lima et al., 2012 ▶). 

In the writhing test, our results showed that the EOVAC and piroxicam (non-selective cyclooxygenase inhibitor) as a positive control significantly reduced writhing response. This model of visceral nociception is a typical model of inflammatory pain and also accepted as a screening method for the assessment of analgesic and/or anti-inflammatory properties of new compounds and chemicals (Tjølsen and Hole, 1997 ▶).

Acetic acid promotes the release of prostaglandins, serotonin, and histamine in the peritoneal fluids (Deraedt et al., 1980 ▶). Therefore, the current results suggested that the EOVAC significantly produced inhibitory effect in this inflammatory model of pain, and this effect may be related to its suppressive effect on the biosynthesis pathway of pro inflammatory substances or reduction of endogenous pro-inflammatory substances release.

Therapeutic effects of VAC in PMS syndrome were already well documented (Prilepskaya et al., 2006 ▶). In an open-labeled clinical observation on migrainous women with PMS, the VAC (40 mg/day) was administrated for a 3-month period and the results indicated that VAC could improve preventative management of both menstrual- and non-menstrual-related migraine headaches. 

Furthermore, it was safe and well tolerated by patients (Ambrosini et al., 2012 ▶). 

EOVAC showed a rapid analgesic effect in the tail immersion test. The analgesic effect of EOVAC was started from 15 min after administration. This result suggested that EOVAC had a rapid analgesic effect. This rapid effect of EOVAC may be due to its lipophilic nature. Because of this lipophilic character, the EOVAC allowed to rapidly absorb from injection site and also rapidly penetrated the blood brain barrier and reached the central nervous system (Buchbauer et al., 1993 ▶). Some of constituents of essential oils such as linalool, eugenol, menthol, and thymol can act on the central and peripheral nervous system as ion channel modulators (De Araújo et al., 2011 ▶). 

Previous report showed that major essential oil components of the ripe fruits of Vitex agnus castus varied in dependence of grinding, maturity, distillation period, and method of extraction used (Sorensen and Katsiotis, 2000 ▶). A study on the essential oil obtained from Vitex agnus castus grown in Turkey resulted in the detection of 27 components. This study showed that major components of the oil were 1,8-cineole (24.98%), sabinene (13.45%), α-pinene (10.60%), α-terpinyl acetate (6.66%), and (Z)-β-farnesene (5.40%) (Sarikurkcu et al., 2009 ▶). These data are different (in percentage and composition) from our GC-MS findings.

We observed high concentration of α-pinene,* R*-(+)-limonene, β-caryophyllene, and sabinene in the EOVAC composition. *R*-(+)-limonene and α-pinene demonstrated analgesic effects in different models of pain (Guimarães et al., 2013 ▶). In addition, sabinene (1%) has been reported to act as an anti-inflammatory agent in the crystalline-induced ocular inflammation in rabbit eyes (Sheng and Chiou, 1993 ▶). β-caryophyllene is another component which exists in the EOVAC. This sesquiterpene has a functional non-psychoactive CB_2_ cannabinoid receptor agonistic activity (Gertsch et al., 2008 ▶). Cannabinoid receptor CB_2_ and its selective agonist were accepted as a new pharmacological target for the treatment of pain (Anand et al., 2009 ▶). Moreover, β- caryophyllene has been shown to inhibit inflammation in the mouse model of experimental colitis induced by dextran sulfate sodium (Cho et al., 2006 ▶) and have a local anesthetic effect (Ghelardini et al., 2001 ▶). Caryophyllene oxide and eugenol are the other two active ingredients that we found in our extracted EOVAC. Chavan et al., (2010) ▶ described that caryophyllene oxide has an analgesic activity in both hot plate and acetic acid-induced writhing test in mice and also has an anti-inflammatory action in carrageenan- induced paw edema in Wistar rats. 

Eugenol is a phenylpropene derivative, well known as local anesthetic, analgesic (Park et al., 2009 ▶), and anti-inflammatory agent (Hashimoto et al., 1988 ▶) that is widely used in dentistry. 

Taken all together, we suggest that at least a part of antinociceptive property of EOVAC may depend on its β-caryophyllene, caryophyllene oxide, α-pinene, limonene, eugenol, and sabinene content.

In the toxicity study, using gavage technique for oral administration of EOVAC at the doses of 1, 2, 3, and 5 g/kg revealed that essential oil was not toxic in mice and we didn’t see any mortality. According to these finding, the LD50 value of EOVAC in mice was estimated to be more than 5 g/kg, p.o. which it can be well tolerated by animals.

In conclusion, our results indicate that the EOVAC produced analgesic effect in these models of nociception and this effect seems to be mediated by activation of endogenous opioidergic system and muscarinergic receptors of cholinergic system. In addition, part of antinociceptive activity of Vitex agnus castus essential oil may be due to its anti-inflammatory effect.

## Conflict of Interest

The authors have declared that there is no conflict of interest.
